# Soluble immune factor profiles in blood and CSF associated with *LRRK2* mutations and Parkinson’s disease

**DOI:** 10.1038/s41531-025-01215-5

**Published:** 2025-11-27

**Authors:** Roshni Jaffery, Yuhang Zhao, Sarfraz Ahmed, Jackson G. Schumacher, Jae Ahn, Leilei Shi, Yujia Wang, Yukun Tan, Jiayin Zhang, Ken Chen, Hussein Tawbi, Jian Wang, Michael A. Schwarzschild, Weiyi Peng, Xiqun Chen

**Affiliations:** 1 Department of Biology and Biochemistry, University of Houston, Houston, TX USA; 2 Aligning Science Across Parkinson’s (ASAP) Collaborative Research Network, Chevy Chase, MD USA; 3 Department of Neurology, Mass General Institute for Neurodegenerative Disease, Massachusetts General Hospital, Harvard Medical School, Boston, MA USA; 4 Department of Melanoma Medical Oncology, The University of Texas MD Anderson Cancer Center, Houston, TX USA; 5 Department of Bioinformatics and Computational Biology, The University of Texas MD Anderson Cancer Center, Houston, TX USA; 6 Department of Biostatistics, The University of Texas MD Anderson Cancer Center, Houston, TX USA

**Keywords:** Biomarkers, Immunology, Neurology, Neuroscience

## Abstract

Mutations in *LRRK2*, a leading genetic cause of Parkinson’s disease (PD), are linked to immune dysregulation, but the immune profiles in the periphery and central nervous system (CNS) remain incompletely defined. This study utilized a large cohort of serum samples (*n* = 651) and matched CSF samples (*n* = 129) from *LRRK2* mutation carriers and non-carriers, with and without PD, to assess immune regulators using Luminex immunoassay. After correction for multiple comparisons, *LRRK2* mutations were associated with significantly elevated serum levels of SDF-1 alpha and TNF-RII, while CSF markers such as BAFF, CD40L, and IL-27 were nominally reduced. Regardless of *LRRK2* status, PD was associated with nominally lower levels of inflammatory analytes in CSF, with minimal changes observed in serum. Correlation analyses revealed distinct immune profiles between serum and CSF, suggesting compartmentalized immune responses. These findings highlight immune alterations in *LRRK2* mutation carriers and PD, providing potential serum markers for monitoring immune responses and avenues for mechanistic studies.

## Introduction

Parkinson’s disease (PD) is a common age-related neurodegenerative disorder characterized by resting tremor, rigidity, bradykinesia, and gait instability, along with non-motor symptoms. Although its exact etiology remains unclear, a combination of genetic and environmental factors contributes to disease development^1,2^. Mutations in the leucine-rich repeat kinase 2 (*LRRK2*) gene are the most frequent genetic cause of autosomal-dominant PD and a population-level risk factor. *LRRK2* PD often presents with clinical features indistinguishable from idiopathic PD, though some studies suggest slower disease progression^3,4^. LRRK2 encodes a multifunctional kinase involved in cytoskeletal dynamics, vesicle trafficking, and autophagy, essential for maintaining cellular homeostasis^5^. Pathogenic *LRRK2* mutations increase kinase activity, which is thought to contribute to dopaminergic neurodegeneration; however, the underlying mechanisms remain incompletely understood.

Growing evidence suggests that immune dysregulation contributes to PD pathophysiology^6,7^. Neuroinflammation, characterized by the activation of microglia and astrocytes and infiltration of peripheral immune cells, has been implicated in the progressive neurodegeneration observed in PD^6,7^. Altered cytokine and chemokine have been reported in blood^8–10^ and cerebrospinal fluid (CSF)^11–13^, indicating systemic immune dysregulation.

LRRK2 is expressed in various immune cell types, including microglia, monocytes, T cells, and B cells^14^. Beyond PD, *LRRK2* variants are associated with inflammatory bowel disease and infection susceptibility^15^, supporting its role in immune regulation. Elevated inflammatory mediators have been observed in *LRRK2* mutation carriers^16^, and increased LRRK2 expression in both innate and adaptive immune cells in PD correlates with enhanced cytokine production^17^, suggesting that heightened LRRK2 kinase activity may drive proinflammatory responses^18^.

To further characterize peripheral and central immune dysregulation associated with *LRRK2* mutations and PD, we profiled 65 soluble immune factors, including cytokines, chemokines, growth factors, and receptors/ligands, in serum and CSF from participants enrolled in the LRRK2 Cohort Consortium (LCC). By comparing individuals with idiopathic PD, *LRRK2* PD, asymptomatic *LRRK2* carriers, and non-carrier controls, we sought to identify potential immune-related markers in serum and CSF associated with PD and *LRRK2* mutation status.

## Results

### Generative pre-trained transformer (GPT)-extracted literature overview of cytokines and PD

We performed a GPT-assisted literature review of the 65 cytokines and PD, which includes PD with *LRRK2* mutations. The number of publications about cytokines, LRRK2, and PD has increased dramatically in recent years, peaking in 2022 and remaining at relatively high levels in the following years (Figure S1). While numerous studies have reported significant elevations in cytokines such as IFN gamma, IL-18, TNF-alpha, and IL-1 beta, a substantial number of other investigations have found no significant association between these and other cytokines and PD. This was observed in the “all papers” search (Figure S2), encompassing all studies across various hosts, including humans, animals, and cell lines (Figure S2A), as well as in human studies only (Figure S2B). Our “primary paper” search, excluding reviews, editorials, letters, and pre-prints, showed similar results (Figure S3). Our GPT-assisted search did not separate blood and CSF.

### Characteristics of the participants

Serum analytes were collected from a total of 651 subjects available in the LCC (Table 1A) with an approximate 2:1 ratio in the number of *LRRK2*+ subjects to the number of *LRRK2*- subjects and an approximate 1:2 ratio in the number of PD subjects to the number of non-PD subjects. PD subjects were significantly older than those without a PD diagnosis (*p* < 0.001). There was also a statistically significant difference (*p* = 0.022) in the male-to-female ratios in each group, as there was a greater proportion of females than males in every group except the *LRRK2-/*PD group. Among the 129 subjects who provided CSF (Table 1B), there was a nearly 1:1 ratio in the number of PD subjects to the number of unaffected controls (UC) and in the number of *LRRK2*+ subjects to those without a *LRRK2* mutation. There was a statistically significant age difference (*p* = 0.024) among the groups, with the *LRRK2*+*/*PD group having the oldest subjects compared to the other three groups. There was no significant difference in terms of sex throughout the groups (Table 1B). Table 1C comprises those who had both serum and CSF samples. There was a near 1:1 ratio between PD:UC and *LRRK2*+:*LRRK2-* subjects, and there was no significant difference in the mean age and sex across the four groups in this subset (Table 1C).

**Table 1:** Demographics and features by *LRRK2* and PD status of participants contributing analyzed samples

A	LRRK2-/UC	LRRK2-/PD	LRRK2+/UC	LRRK2+/PD	Total	*p* value
Serum (*n*)	(*n* = 148)	(*n* = 65)	(*n* = 261)	(*n* = 177)	(*n* = 651)	
Age, mean (SD)	53.2 (15.3)	62.8 (11.2)	50.8 (14.8)	65.9 (11.2)	56.7 (15.2)	<0.001
Gender						
Female	96 (64.9%)	29 (44.6%)	139 (53.3%)	92 (52.0%)	356 (54.7%)	0.022
Male	52 (35.1%)	36 (55.4%)	122 (46.7%)	85 (48.0%)	295 (45.3%)	
Sample cohort						
33084	66 (44.6%)	25 (38.5%)	161 (61.7%)	73 (41.2%)	325 (49.9%)	<0.001
33725	82 (55.4%)	40 (61.5%)	100 (38.3%)	104 (58.8%)	326 (50.1%)	
**B**						
CSF (*n*)	(*n* = 32)	(*n* = 34)	(*n* = 39)	(*n* = 24)	(*n* = 129)	*p* value
Age, mean (SD)	54.8 (14.1)	58.1 (11.4)	51.7 (15.7)	62.1 (11.1)	56.1 (13.9)	0.024
Gender						
Female	18 (56.2%)	10 (29.4%)	19 (48.7%)	12 (50.0%)	59 (45.7%)	0.143
Male	14 (43.8%)	24 (70.6%)	20 (51.3%)	12 (50.0%)	70 (54.3%)	
Sample cohort						
32774	11 (34.4%)	3 (8.8%)	2 (5.1%)	2 (8.3%)	18 (14.0%)	0.004
33826	21 (65.6%)	31 (91.2%)	37 (94.9%)	22 (91.7%)	111 (86.0%)	
**C**						
Serum and CSF (*n*)	(*n* = 20)	(*n* = 32)	(*n* = 35)	(*n* = 18)	(*n* = 105)	*p* value
Age, mean (SD)	54.2 (15.9)	58.6 (11.4)	53.5 (15.4)	59.6 (10.9)	56.2 (13.7)	0.243
Gender						
Female	12 (60.0%)	10 (31.2%)	18 (51.4%)	9 (50.0%)	49 (46.7%)	0.185
Male	8 (40.0%)	22 (68.8%)	17 (48.6%)	9 (50.0%)	56 (53.3%)	

Sample size, mean age (SD), sex, and *p* value by group status for (A) serum, (B) CSF, and (C) matching serum and CSF samples. A and B also list group status according to sample cohort.

### Presence of soluble immune factors in serum and CSF samples

A total of 65 analytes were quantified from the four groups of serum and CSF samples. In serum, 22 out of the 65 analytes were detectable in over 50% of the samples. These analytes included APRIL (a proliferation-inducing ligand), BLC (B lymphocyte chemoattractant, also termed as chemokine ligand 13 CXCL13), CD30, ENA-78 (epithelial neutrophil-activating protein 78, also known as CXCL5), Eotaxin, Eotaxin-2, HGF (hepatocyte growth factor), IL-16, IL-18, IL-2R (IL-2 receptor, also known as CD25), IL-7, IP-10 (IFN-γ inducible protein, also termed as CXCL10), MCP-1 (monocyte chemoattractant protein-1, also termed as CCL2), MCP-2 (monocyte chemoattractant protein-2 also known as CCL8), MDC (macrophage-derived chemokine, also termed as CCL22), MIF (macrophage migration inhibitory factor), MMP-1 (matrix metalloproteinase-1), SCF (stem cell factor), SDF-1 alpha (stromal cell-derived factor-1 alpha, also known as CXCL12), TNF-RII (TNF-related apoptosis-inducing ligand, CD253), Tweak (tumor necrosis factor-like weak inducer of apoptosis), and VEGF-A (vascular endothelial growth factor A). In contrast, 63 analytes were detectable in more than 50% of the CSF samples, except for IL-5 and TRAIL (TNF-related apoptosis-inducing ligand, CD253), which have detection rates of 49.6% and 36.4%, respectively, in CSF samples. The means of tested analytes and their detection rates in percentages in serum and CSF samples were summarized in Figure S4. A broad range of soluble immune factors in CSF supports the potential involvement of CNS immune responses in this set of samples. It provides a rationale for identifying differentially expressed analytes in subjects with PD and *LRRK2* mutations.

### Differentiated soluble immune factors in serum and CSF in *LRRK2* mutation carriers

Although exploratory, we calculated *p* values both with (*p* adj) and without (*p*) adjustment for multiple comparisons for all analyses. We highlight findings that remain significant after correction (*p* adj ≤ 0.05), while also noting all associations with nominal significance (*p* ≤ 0.05) to include all potential immune factors associated with PD or *LRRK2* mutations. All original data and analyses are available at https://zenodo.org/records/14424178.

**Fig. 1:**
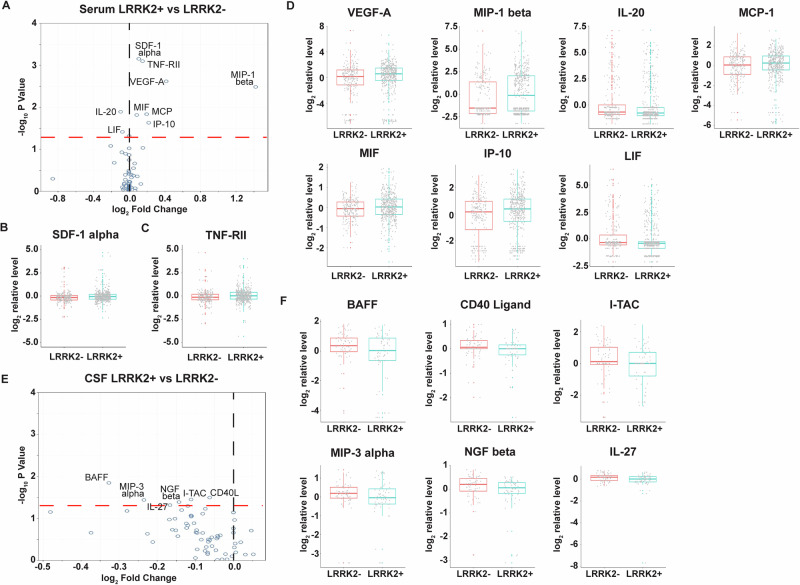
Changes of soluble immune factors in serum and CSF samples of LCC participants carrying *LRRK2* mutations. **A** Volcano plot of serum analytes comparing *LRRK2*+ (*n* = 438) and *LRRK2*- (*n* = 213) participants. **B**–**D** Batch-corrected concentrations of SDF-1 alpha, TNF-RII, VEGF-A, MIP-1 beta, MCP-1, MIF, IP-10, IL-20, and LIF in serum of *LRRK2*+ and *LRRK2*- participants. **E** Volcano plot of CSF analytes comparing *LRRK2*+ (*n* = 63) vs *LRRK2*- (*n* = 66) precipitants. **F** Batch-corrected concentrations of BAFF, CD40-Ligand, I-TAC, MIP-3 alpha, NGF beta, and IL-27 in CSF comparing *LRRK2*+ and *LRRK2*- participants. The volcano plots illustrate the data normalized to the mean. The black and red dashed line represents “log_2_ Fold Change” of 0 and “-log_10_ *P* Value” of 1.3, respectively. The boxplots illustrate the median (represented by the horizontal line within the box), and the box represents the upper and lower quartiles. Robust linear regression adjusting for age, sex, sample cohort, and PD status.

To identify changes in soluble immune regulators associated with *LRRK2* mutations, we compared serum and CSF results from subjects carrying *LRRK2* mutations (*n* = 438) with those from non-carriers (*n* = 213), irrespective of PD status. After adjusting for age, sex, sample cohort, and PD status, multivariable linear regression analysis identified seven elevated and two reduced immune factors with *p* value < 0.05 in the *LRRK2* mutation carrier group (Fig. 1A). Notably, *LRRK2* mutation carriers demonstrated an increase in SDF-1 alpha, a stromal cell-derived chemokine in serum, compared to non-carriers (*p* = 0.0007). The difference is statistically significant after adjusting for multiple comparisons (*p* adj = 0.026) (Fig. 1B). Another central immune regulator, TNF-RII, was found to have a higher concentration in *LRRK2* mutation carriers compared to non-carriers, and a significant difference was observed between the two groups before and after multiple comparison adjustments (*p* = 0.0008, *p* adj = 0.026) (Fig. 1C).

Compared to non-carriers, additional elevated analytes in serum of *LRRK2* mutation carriers included VEGF-A (*p* = 0.002), MIP-1 beta (*p* = 0.003), MCP-1 (*p* = 0.015), MIF (*p* = 0.015), and IP-10 (*p* = 0.024). In contrast, IL-20 (*p* = 0.013) and LIF (leukemia inhibitory factor, *p* = 0.038) were reduced in the serum of *LRRK2* mutation carriers compared to non-carriers (Fig. 1D). However, *p* values for these analytes were over 0.05 after multiple comparison adjustment.

In contrast to serum, *LRRK2* mutations were only associated with moderately reduced soluble immune markers in CSF. Analysis of 129 samples revealed six reduced analytes in subjects carrying *LRRK2* mutations (*n* = 63) compared with those who were non-carriers (*n* = 66), regardless of PD status (Fig. 1E). These analytes, which included BAFF (B cell-activating factor; *p* = 0.014), CD40-Ligand (*p* = 0.032), I-TAC (IFN-inducible T cell alpha chemoattractant, also known as CXCL11; *p* = 0.035), MIP-3 alpha (*p* = 0.036), NGF beta (nerve growth factor-beta; *p* = 0.041), and IL-27 (*p* = 0.048), were all reduced (Fig. 1F). However, none of these differences remained statistically significant after adjusting for multiple comparisons.

**Fig. 2:**
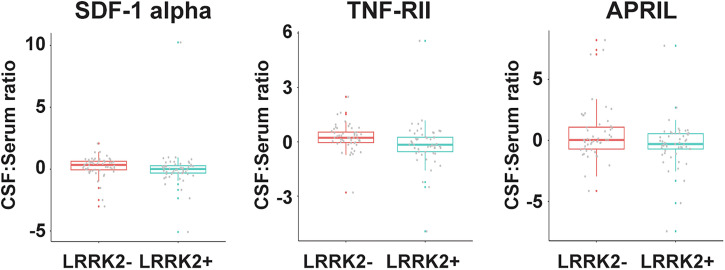
Altered CSF:serum ratios of soluble immune factors comparing *LRRK2*+ and *LRRK2*- participants. CSF:serum ratios for SDF-1 alpha, TNF-RII, and APRIL in *LRRK2*+ (*n* = 53) and *LRRK2*- (*n* = 52) participants. The boxplots illustrate the median (represented by a horizontal line within the box), and the box represents the upper and lower quartiles. Robust linear regression adjusting for age, sex, sample cohort, and PD status.

We calculated CSF:serum ratios of all analytes from the subset of matching 105 serum and CSF samples, which had well-balanced numbers of *LRRK2* mutation carriers (*n* = 53) vs non-carriers (*n* = 52). CSF:serum ratios for TNF-RII and SDF-1 alpha were lower in *LRRK2* carriers than non-carriers (*p* = 0.005 and 0.007, respectively; *p* adj = 0.0107 for both), which were most likely driven by changes in the levels of the two analytes in serum. Additionally, CSF:serum ratio for APRIL, another member of the TNF superfamily, was lower in *LRRK2* carriers than non-carriers in this subset (*p* = 0.031) (Fig. 2). These changes were not statistically significant after multiple comparison adjustment. For the remaining analytes where CSF:serum ratios could be calculated, no differences were observed between *LRRK2* mutation carriers and non-carriers.

### Differentiated soluble immune factors in serum and CSF in PD subjects

**Fig. 3:**
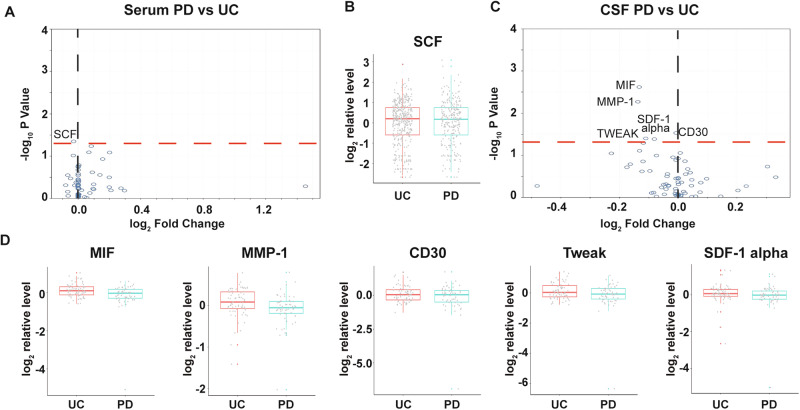
Changes of soluble immune factors in serum and CSF samples of LCC participants with PD. **A** Volcano plot of serum analytes comparing PD (*n* = 182) and unaffected controls (*n* = 409). **B** Batch-corrected concentrations of SCF in serum of PD subjects and unaffected controls. **C** Volcano plot of CSF analytes PD (*n* = 58) vs unaffected controls (*n* = 71). **D** Batch-corrected concentrations of MIF, MMP-1, CD30, Tweak, and SDF-1 alpha in CSF of PD subjects and unaffected controls. The volcano plots illustrate the data normalized to the mean. The black and red dashed line represents “log_2_ Fold Change” of 0 and “-log_10_ *P* Value” of 1.3, respectively. The boxplots illustrate the median (represented by the horizontal line within the box), and the box represents the upper and lower quartiles. Robust linear regression adjusting for age, sex, sample cohort, and *LRRK2* mutation.

We compared serum and CSF results from PD (*n* = 242) and control subjects (*n* = 409), irrespective of *LRRK2* mutations. Surprisingly, there were no dramatic differences in serum concentrations of all the analytes, except for a marginally lower SCF in PD than in the control group (*p* = 0.045) (Fig. 3A, B). In CSF, no analytes were elevated. Concentrations of MIF (*p* = 0.002), MMP-1 (*p* = 0.005), CD30 (*p* = 0.030), Tweak (*p* = 0.040), and SDF-1 alpha (*p* = 0.042) were lower in PD (*n* = 58) as compared to control subjects (*n* = 71) (Fig. 3C, D). However, none of these differences remained statistically significant after adjusting for multiple comparisons.

**Fig. 4:**
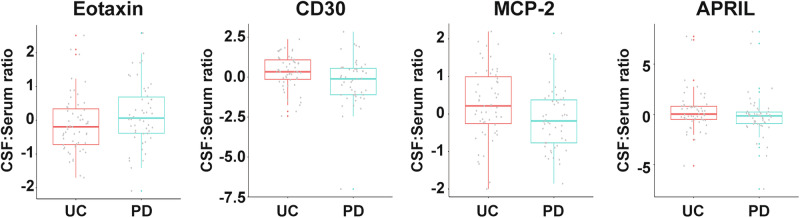
Altered CSF:serum ratios of soluble immune factors in PD subjects compared with unaffected controls. CSF:serum ratios for Eotaxin, CD30, MCP-2, and APRIL in PD subjects (*n* = 50) and UC subjects (*n* = 55). The boxplots illustrate the median (represented by a horizontal line within the box), and the box represents the upper and lower quartiles. Robust linear regression adjusting for age, sex, sample cohort, and *LRRK2* mutation.

In PD subjects from the subset with matching serum and CSF, the CSF:serum ratios for CD30 (*p* = 0.002), MCP-2 (*p* = 0.010), and APRIL (*p* = 0.021) were lower, while Eotaxin was higher (*p* = 0.030) compared to the control (Fig. 4) before multiple comparison adjustment.

#### *LRRK2* PD-associated changes in immune markers

To identify potential inflammatory and immune markers associated with *LRRK2* and PD, we performed four-group comparisons using the robust linear regression models, adjusting for age, sex, and sample cohort. In serum, compared with the idiopathic PD (*LRRK2-*/PD) group, the *LRRK2*+/PD group had lower CD30 levels (*p* = 0.020). Compared with *LRRK2* mutation carriers without PD (*LRRK2*+/UC), *LRRK2*+/PD groups had lower SCF (*p* = 0.025) (Table 2A). There were no significant differences in serum concentrations of SDF-1 alpha and TNF-RII between PD subjects with *LRRK2* mutations and those without. The overall difference in SDF-1 alpha between *LRRK2*+ and *LRRK2*- groups (effect size = 0.1135) in Fig. 1 appears to be primarily attributable to the difference between *LRRK2*+ and *LRRK2*- subjects within the UC group (effect size = 0.0416, *p* = 0.0008). Additionally, we did not observe any significant differences in serum concentrations of SDF-1 alpha and TNF-RII between *LRRK2* mutation carriers with PD and those without (Table 2A).

**Table 2:** Altered analytes in *LRRK2*+ PD subjects from 4-group comparisons

A Serum *n* = 651			
Analyte	Comparison	Estimate	*p* value
	LRRK2+/PD vs LRRK2-/PD		
CD30		−0.334	0.020
SDF-1 alpha		0.061	0.296
TNF-RII		0.085	0.264
	LRRK2+/PD vs LRRK2+/UC		
SCF		−0.254	0.025
SDF-1 alpha		−0.006	0.888
TNF-RII		−0.065	0.250
**B CSF** ***n*** **=** **129**			
Analyte	Comparison	Estimate	*p* value
	LRRK2+/PD vs LRRK2-/PD		
SDF-1 alpha		−0.178	0.011
CD40-Ligand		−0.203	0.045
BAFF		−0.494	0.050
TNF-RII		−0.023	0.817
	LRRK2+/PD vs LRRK2+/UC		
SDF-1 alpha		−0.188	0.007
MIF		−0.205	0.028
TNF-RII		−0.046	0.647
**C CSF:serum ratio** ***n*** **=** **105**			
Analyte	Comparison	Estimate	*p* value
	LRRK2+/PD vs LRRK2-/PD		
TNF-RII		-0.515	0.008
SDF-1 alpha		-0.447	0.013
IL-16		0.436	0.042
LIF		0.342	0.049
	LRRK2+/PD vs LRRK2+/UC		
SDF-1 alpha		-0.258	0.149
TNF-RII		-0.350	0.068

A: Full set of serum samples. B: Full set of CSF samples. C: CSF:serum ratios. Robust linear regression adjusting for age, sex, and sample cohort.

In CSF, compared to the *LRRK2*-/PD group, the *LRRK2*+/PD group had lower SDF-1 alpha levels (*p* = 0.011), which is the opposite of what was observed in the serum. Of note, there was no difference between *LRRK2*+/UC and *LRRK2*-/UC (*p* = 0.678). CD40L (*p* = 0.045) and BAFF (*p* = 0.050) were also lower in the *LRRK2*+/PD group (Table 2B). SDF-1 alpha (*p* = 0.007) was lower, in addition to MIF (*p* = 0.028), in the *LRRK2*+/PD group compared to the *LRRK2*+/UC group (Table 2B). Reduced TNF-RII levels in CSF were also observed, comparing the *LRRK2*+/PD group to the *LRRK2*-/PD group, but the magnitude of the difference was minor (Table 2B). These results are overall consistent with the trends of lower CSF markers observed in the *LRRK2*+ vs *LRRK2*- and PD vs UC groups, as shown in Figs. 1E and 3C.

In terms of the CSF:serum ratios of the analytes, the *LRRK2*+/PD group had significantly decreased concentration ratios of TNF-RII (*p* = 0.008), SDF-1 alpha (*p* = 0.013), IL-16 (*p* = 0.042) and LIF (*p* = 0.049) compared to *LRRK2*-/PD. No significant changes were observed between the *LRRK2*+/PD and *LRRK2*+/UC groups in concentration ratios of TNF-RII, SDF-1 alpha, or other analytes (Table 2C).

None of the analyte differences in the four-group comparisons remained statistically significant after adjusting for multiple comparisons.

### Correlations between serum and CSF soluble immune factors

**Fig. 5:**
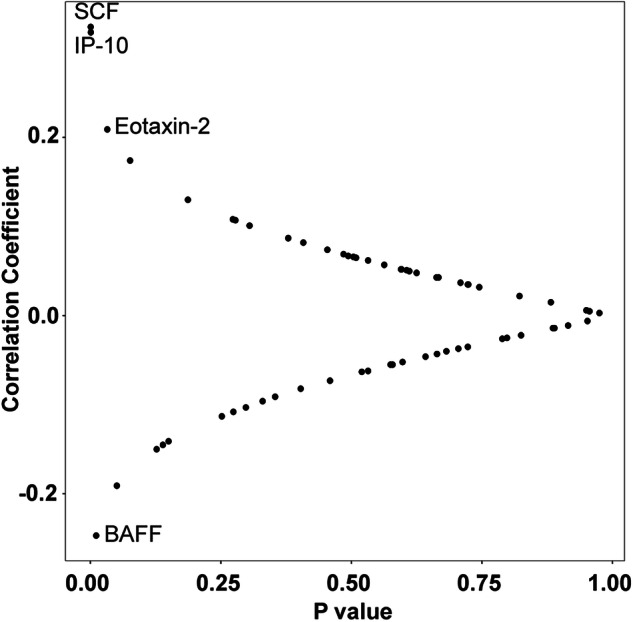
Correlation between serum and CSF concentrations of soluble immune factors. Spearman correlation coefficient test (*n* = 105).

Given that the altered analytes in serum and CSF related to *LRRK2* mutations and PD were largely non-overlapping or had an inverse relationship, we analyzed the correlation between serum analytes and CSF analytes in the subset of matching serum and CSF samples using the Spearman correlation coefficient. Four out of 65 analytes showed a statistically significant correlation between serum and CSF. As shown in Fig. 5, SCF, IP-10, and Eotaxin-2 in serum and CSF were positively correlated (*p* = 0.0007, 0.001, and 0.033, respectively). Correlations of SCF and IP-10 between serum and CSF were still significant after multiple comparison adjustments (*p* adj = 0.031 for both). Serum and CSF BAFF concentrations were negatively correlated (*p* = 0.01).

## Discussion

Our study using the LCC as a discovery cohort revealed that *LRRK2* mutations were associated with increased immune markers in serum, not CSF, whereas PD was associated with nominally decreased immune markers in CSF, not serum. Specifically, subjects harboring pathogenic mutations in the *LRRK2* gene show increased production of immune regulators in serum. SDF-1 alpha and TNF-RII, in particular, have been identified as potential serum markers for immune dysregulation in *LRRK2* mutation carriers, though not to the extent of differentiating *LRRK2*+/PD and *LRRK2-*/PD or *LRRK2*+/UC and *LRRK2-*/UC.

SDF-1 alpha, also known as CXCL12, is a potent chemotactic protein produced by bone marrow stromal cell lines. Through its two receptors, CXCR4 and CXCR7, SDF-1 alpha acts as a key homeostatic chemokine regulating embryogenesis, hematopoiesis, and angiogenesis^19^. It promotes migration, proliferation, and maturation of hematopoietic progenitor cells, endothelial cells, and leukocytes. SDF-1 alpha expression is elicited in primary and secondary lymphoid organs as a part of the homeostatic mechanism regulating immune cell development and trafficking^19^. It is also involved in regulating inflammation, playing a vital role in wound healing and tissue repair in inflammatory diseases^19^. SDF-1 alpha has been reported to promote cancer and arthritic diseases; however, it has also been shown to be cardioprotective by promoting stem cell homing, angiogenesis, and remote ischemic conditioning^20^. In the CNS, SDF-1 alpha signaling promotes the proliferation, differentiation, and migration of neural precursor cells and mediates axonal elongation and branching after cerebral ischemia^21^. SDF-1 alpha can promote microglial phagocytosis of amyloid beta and be neuroprotective in Alzheimer’s disease (AD)^19,22^. More recent studies have shown that SDF-1 alpha may also be proinflammatory and mediates alpha-synuclein-induced microglia accumulation^23^. We were unable to find evidence of serum SDF-1 alpha in *LRRK2* mutation carriers in the literature. A study showed a higher level of SDF-1 alpha in the CSF of asymptomatic *LRRK2* mutation carriers, but the difference was not statistically significant^24^. In PD patients, blood SDF-1 alpha was found to be significantly higher than in control subjects in a smaller cohort^25^. An upregulation of the CXCL12/CXCL4 signaling pathway has been shown to be involved in the loss of dopaminergic neurons in animal models^26^. These findings suggest that SDF-1 alpha may be involved in disease- or model-specific immune mechanisms contributing to neurodegeneration. Our findings of a significant increase in blood SDF-1 alpha in *LRRK2* mutation carriers and nominal decreases in CSF SDF-1 alpha in *LRRK2* PD suggest a dysregulated gradient across the BBB, which may compromise its homeostasis and function in the CNS. However, whether the altered SDF-1 alpha is mechanistically involved in *LRRK2* mutations and increased PD risk requires further investigation.

TNF-alpha has been reported to be significantly higher in the CSF and serum of asymptomatic *LRRK2* mutation carriers compared to healthy controls^16,27^. TNF-alpha binds to TNF-RI and TNF-RII, playing a crucial role in both innate and adaptive immune responses. While TNF-RI is ubiquitously expressed in almost all cell types and predominantly mediates proinflammatory responses, TNF-RII has been shown to be expressed predominantly on regulatory T cells (Tregs)^28,29^. Through the immune-modulatory function of Tregs, TNF-RII promotes tissue homeostasis and regeneration^30^. Mechanistic studies indicate that TNF-RII activation is neuroprotective. In a mouse model of AD, it mitigated cognitive deficits and neuropathology by decreasing amyloid β production and enhancing its clearance by glial cells^31,32^. Soluble TNF-RII, released via proteolytic cleavage of the transmembrane receptors, binds to and effectively neutralizes TNF-alpha, acting as a negative regulator of TNF-alpha signaling^29,30^. Clinical studies have demonstrated a deficiency in TNF-RII signaling in various autoimmune diseases^28,33^. Levels of soluble TNF-RII, however, are elevated in the serum of patients with multiple sclerosis^34^, likely reflecting a compensatory mechanism that mitigates excessive TNF-alpha activity through its decoy receptor function. With its central role in regulating inflammation, TNF-RII has been increasingly recognized as a potential therapeutic target for inflammatory conditions such as rheumatoid arthritis and inflammatory bowel disease^35,36^. The elevated serum soluble TNF-RII levels observed in our study may reflect a compensatory mechanism activated to maintain immune homeostasis in *LRRK2* mutation carriers, providing new directions for understanding immune dysregulation in *LRRK2*-related disorders.

Other *LRRK2* mutations-related changes in serum that did not survive multiple comparison adjustment include increases in VEGF-A, which promotes angiogenesis and growth of solid tumors, MIP-1 beta, which is produced mainly by macrophages and monocytes and has been shown to orchestrate protective responses against various viral infections^37,38^, MCP-1, which promotes the migration of inflammatory cells and is a key protein in tumor development^39^, MIF, which sustains the survival and function of macrophages and drives inflammation^40,41^, and IP-10, which controls cell growth and development including tumor cells growth and angiostasis^42^. Among the reduced serum analytes, IL-20 induces the proliferation of epithelial cells and the production of proinflammatory factors^43^. LIF is a pleiotropic cytokine that maintains the homeostasis and regeneration of multiple tissues^44^. The nominal changes in these factors, together with significantly elevated SDF-1 alpha and TNF-RII, indicate enhanced peripheral immune activation, promoting leukocyte trafficking, chemotaxis, and regulatory signaling.

Compared with changes identified in serum, *LRRK2* mutations are associated with a completely different profile of immune and inflammatory factors in CSF, which showed moderately and non-significantly reduced signals related to the regulation of B cell selection and survival (BAFF)^45^, T–B cell communications (CD40L through interaction with CD40)^46^, immune cell recruitment (I-TAC), dendritic cell trafficking (MIP-3 alpha), neural development and survival (NGF beta), and inhibition of inflammation (IL-27)^47^. Collectively, the alterations in these immune factors may indicate suppressed central adaptive immunity, particularly affecting B cell survival and T–B cell interactions, which warrant further validation.

The overall non-significant findings in PD subjects in this large cohort of samples were unexpected. Out of 65 immune markers analyzed, only SCF was nominally reduced in serum in PD subjects compared to those in healthy controls. SCF is a survival and growth factor for hematopoietic stem and progenitor cells. Previous studies have shown increased or decreased levels of SCF in PD^48,49^. The nominally reduced signals in CSF include MIF, MMP-1, which is involved in extracellular matrix remodeling and regulating proinflammatory cytokines^50^, CD30, a member of the TNF receptor family that mediates pro-survival signals^27^, Tweak, a TNF superfamily member that can stimulate inflammatory cytokines and determine synaptic function^51^, and SDF-1 alpha. Literature evidence is limited for these immune markers in the CSF of PD patients. Previous studies using serum samples have found significantly lower levels of MMP-1^52^ and higher levels of MIF in PD patients^53^. The exact roles of these immune regulators in PD have yet to be elucidated.

The non-overlapping patterns between changed analytes in serum and CSF, comparing either *LRRK2+* vs *LRRK2-* or PD vs UC, revealed overall disconnection between serum and CSF in the LCC. This is supported by correlation analysis, which shows no positive correlations between serum and CSF for all but two of the analytes after multiple comparison adjustment (SCF and IP-10). The finding aligns with previous studies that report a poor correlation between peripheral and central inflammatory markers. A study in PD patients found correlations between serum and CSF inflammatory markers for only a subset of analyzable inflammatory markers. Similarly, in healthy control subjects from the same study^54^, only a small subset of cytokines, such as IL-4 and leptin, showed significant serum–CSF correlations. Another study found that peripheral and CSF cytokine profiles in PD were not closely related^48^. A systematic meta-analysis of 21 studies (*n* = 1679 paired samples) across neurological and psychiatric disorders showed a low pooled correlation between serum and CSF cytokine levels^55^. The disconnection suggests that these analytes may be produced in different compartments and that the integrity of the blood-brain barrier (BBB) may not be severely compromised by either *LRRK2* mutations or PD. Active transport mechanisms across the BBB may also contribute to maintaining the differences between serum and CSF. Additionally, differences in the kinetics of cytokine and chemokine production and clearance between serum and CSF may result in discrepancies in their levels at the time of sampling.

Numerous studies have demonstrated elevated levels of various immune markers linked to either *LRRK2* or PD, whereas some reports indicate inconsistencies in immune marker expression^8–14,16,56–58^. This highlights the complexity of immune and inflammatory-related responses associated with *LRRK2* and PD. Our study did not find changes in TNF-alpha, IL-6, IL-1 beta, IL-10, IFN gamma, or IL-18 in either serum or CSF, comparing *LRRK2* or PD status. The discrepancies across studies could stem from various factors, including clinical heterogeneity among participants, variations in inclusion criteria, differences in sample sizes, and inconsistencies in sample processing or analytical techniques. Additionally, the timing of sample collection, differences in disease stage or progression among subjects, and potential environmental or other genetic factors may also contribute to the mixed results from different studies.

Our study has limitations. A methodological consideration is our kit selection, which offers a broad coverage of 65 key immune factors relevant to both central and peripheral immune responses while maintaining compatibility with limited sample volumes. However, the panel is not exhaustive, and there may be potentially relevant immune factors that are outside its detection scope. Additionally, although we have adjusted for relevant covariates across groups in our analyses, including age and sex, and PD status or *LRRK2* mutation when applicable, unmeasured confounders may influence our results, such as disease duration, sampling timing, and sample storage duration, PD and other medications especially immune-modulating medications, or medical background that is key in this study. Other limitations inherited from the LCC include possible misclassification of PD and UC, given that there was no pathological diagnosis. Furthermore, sample selection bias cannot be excluded. Future studies should validate these findings in an independent cohort. Finally, functional valuation studies should be conducted to explore mechanistic associations between candidate markers and *LRRK2* mutations and/or PD, especially for SDF-1 alpha and TNF-RII.

Our study provides comprehensive immune profiles associated with *LRRK2* mutations and PD in serum and CSF in a large cohort. We identified SDF-1 alpha and TNF-RII as serum markers of immune dysregulation in *LRRK2* mutation carriers, offering a new framework for understanding PD pathogenesis. The disconnection between peripheral and central immune signatures in both *LRRK2* carriers and PD patients suggests distinct mechanistic pathways underlie compartment-specific immune dysregulation. Future investigations are needed to validate these immune markers in independent cohorts and to dissect how they functionally relate to LRRK2 biology and/or PD.

## Methods

### Summary of reported literature on cytokine study using GPT models

To analyze the associations between cytokine concentrations and PD, including those carrying mutations of *LRRK2*, we used a GPT model to extract cytokine-related information from the literature^59^. GPT models are a type of large language model (LLM) that learns patterns and structures from existing data, such as text and images, using deep learning and applying them to analyze and generate content^60^. Leveraging OpenAI’s 4o GPT model, we extracted cytokine information through the following steps: the most relevant papers were retrieved from the PubMed Central (PMC) database through an application programming interface (API) using keywords: (cytokine name) AND (LRRK2 AND Parkinson) OR (LRRK2) OR (Parkinson). The search was limited to a maximum of 50 papers per cytokine. Publication years were retrieved through the PubMed API. Full texts, excluding references, were processed by the subsequent GPT model.

### GPT prompt engineering and postprocessing

We structured the instructions for the GPT model into two categories: custom prompts and system prompts. In custom prompts, instructions were provided to confirm the presence of the cytokine of interest, define disease context, host type, measurement site, association with *LRRK2*, mutation variants, association with PD, and, most importantly, supportive evidence directly extracted from the literature. The system prompt ensured the quality and consistency of the model response by providing definitions of *LRRK2* mutation and its association, as well as a list of alternative names for cytokines. Scores of 1 were given to positive associations between higher cytokine concentrations and PD or the presence of *LRRK2* mutation, -1 to negative associations, and 0 to no associations. The GPT model inferred the associations based on texts in the literature. The model was instructed to assume a positive association if 1) a cytokine’s concentration is higher in PD or PD model hosts if provided, or 2) in the presence of the cytokine, the odds ratio between PD and non-PD is greater than 1 and the lower bound of confidence interval of greater than 1; or) the inhibition of the cytokine is associated with increased risks of PD. We included quality control steps^61^ in custom prompts to verify the extracted content against the literature and make necessary corrections. The model’s performance was improved through iterative validation of the extracted responses, with prompt modifications applied based on the validation results. Each iteration of manual evaluation of model accuracy involved randomly selecting 10 to 30 PubMed IDs (PMIDs) and validating the model’s responses for cytokine extracted, disease type, support for *LRRK2* mutation, support for association with PD, host, and sites. Entries with no cytokine information found were removed from the analysis. For association analysis, two entries with disease types equal to traumatic brain injury (TBI) and depression in PD (dPD) were removed due to irrelevance.

### Human samples

This project utilized samples from the LCC provided by the Michael J. Fox Foundation (MJFF). LCC is a large dataset established in 2009 with coordination and funding from the Michael J. Fox Foundation for Parkinson’s Research (MJFF). The LCC comprises individuals diagnosed with idiopathic PD (*LRRK2*-/PD), unaffected non-carrier controls (*LRRK2*-/UC), carriers of pathogenic *LRRK2* mutations who also have PD (*LRRK2*+/PD), and *LRRK2* mutation carriers without PD symptoms (*LRRK2*+/UC). The specific *LRRK2* mutations found in the cohort of serum samples include major variants G2019S (69%), R1441G/C/H (13%), and minor variants (<2% each) S1228T, R793M, R1325Q, Q930R, N1437H, L1795F, L1114L, I810V, I2020T, G2385R, and C228S. Additional details about the LCC have been published and can be accessed at michaeljfox.org/data-sets and michaeljfox.org/lccinvestigators.

We received a total of 651 serum samples and 129 CSF samples in 4 sample cohorts from the LCC. Samples were stored at -80°C until analyzed.

This study uses de-identified samples and clinical data from the LCC, and qualifies as exempt human subjects research under Title 45 Code of Federal Regulations (CFR) 46; accordingly, no additional ethical approval was required.

### Luminex-immunoassay

The levels of 65 cytokines, chemokines, growth factor targets, and soluble receptors in serum and CSF samples were determined by the ProcartaPlex human immune monitoring kit (EPX650-10065-901, ThermoFisher Scientific, Waltham, MA) on a Luminex-200 system according to the manufacturer’s instructions. The samples were processed in prearranged batches, each containing approximately balanced numbers of samples from different groups. Each serum or CSF sample was aliquoted at a volume of 25 microliters, diluted 1:2 with PBS, and measured in duplicate for cytokine analysis.

The human immune monitoring kit includes 65 pre-determined cytokines, chemokines, and growth factor targets, and soluble receptors, namely G-CSF (granulocyte colony-stimulating factor, also known as colony-stimulating factor 3 (CSF-3)), GM-CSF (granulocyte-macrophage colony-stimulating factor), IFN alpha, IFN gamma, IL-1 alpha, IL-1 beta, IL-2, IL-3, IL-4, IL-5, IL-6, IL-7, IL-8 (also known as CXCL8), IL-9, IL-10, IL-12 (also termed as IL-12p70), IL-13, IL-15, IL-16, IL-17A (also termed as CTLA-8), IL-18, IL-20, IL-21, IL-22, IL-23, IL-27, IL-31, LIF, M-CSF (macrophage colony-stimulating factor), MIF (macrophage migration inhibitory factor), TNF-alpha, TNF-beta, and TSLP (thymic stromal lymphopoietin). The compatible chemokines include BLC, ENA-78, Eotaxin (also known as CCL11), Eotaxin-2 (also termed as CCL24), Eotaxin-3 (also known as CCL26), Fractalkine (also termed as CX3CL1), Gro-alpha (also known as CXCL1), IP-10, I-TAC, MCP-1, MCP-2, MCP-3 (monocyte chemoattractant protein-3 also known as CCL7), MDC, MIG (monokine induced by IFN-γ, also known as CXCL9), MIP-1 alpha (macrophage inflammatory protein-1 alpha, also termed as CCL3), MIP-1 beta (also known as CCL4), MIP-3 alpha (also termed as CCL20), SDF-1 alpha. The compatible growth factors include FGF-2 (fibroblast growth factor-2), HGF, MMP-1, NGF beta, SCF, and VEGF-A (vascular endothelial growth factor). The compatible soluble receptors are APRIL, BAFF, CD30, CD40L (also known as CD154), IL-2R, TNF-RII, TRAIL, and Tweak.

### Data processing and statistical analysis

The Luminex Immunoassay was performed blinded. The data was collected and sent back to MJFF for unblinding. For data processing, the lower-than-lower limit of detection values was set to 0, while the higher-than-higher limit of detection value was set to 100,000. For each analyte, distributional characteristics were assessed using the Shapiro-Wilk test and visual inspections (e.g., histograms). Outliers were defined using the IQR method, as values below the first quartile minus 1.5 times the interquartile range or above the third quartile plus 1.5 times the interquartile range. The number of outliers for each analyte is provided in the Supplementary Material. The log2-transformed data were used for all analyses to address outliers and non-normal distributions. A small constant of 1 was added to all analyte values (i.e., log2[value + 1]) to avoid undefined values for zero measurements. Batch effects were investigated using clustered heatmaps and corrected through batch mean centering. For demographic variables, continuous variables (e.g., age) were summarized using means and standard deviations; categorical variables (e.g., sex) were summarized using counts and frequencies. Kruskal-Wallis test and Fisher’s exact test were used to compare the continuous and categorical data, respectively.

While log2-transformation improved the distributional symmetry of many analytes, some remained non-normally distributed and included potential outliers. To address this, we applied multivariable robust linear regression models, which limit the influence of outliers and are more reliable when residuals deviate from normality, to assess the associations between analyte levels, *LRRK2* mutations, and PD status. For each analyte, we modeled log2-transformed values as the outcome and included *LRRK2* mutation and PD status as primary independent variables in a single regression model. All models were adjusted for age, sex, and sample cohort to account for potential confounders, based on their known or plausible associations with *LRRK2*, PD status, or the analytes. Group comparisons (e.g., *LRRK2*+ vs *LRRK2*-, PD vs UC) were based on model-estimated contrasts, which provide estimates adjusted for the other variables in the model.

A supplementary analysis was also performed using a combined four-group variable representing the joint *LRRK2*/PD status as the independent variable in the model. Correlations were evaluated using the Spearman correlation coefficient. Given that the study was exploratory, we assessed *p* values with (denoted as “*p* adj”) and without (denoted as “*p*”) multiple comparisons adjustment, using Benjamini and Hochberg’s approach, which controls the false discovery rate (FDR). The statistical analyses were conducted using R software (R Development Core Team). All tests were two-sided, and *p* values ≤ 0.05 were considered statistically significant. Results that did not meet significance thresholds after correction are presented as hypothesis-generating, noting they require further validation.

## Supplementary information

Supplementary Information

## Data Availability

Data used in the preparation of this article is openly available to qualified researchers. Demographic data were obtained from the LRRK2 Cohort Consortium Database (www.michaeljfox.org/data-sets). All new data obtained from this study have been deposited in the LRRK2 Cohort Consortium Database and are located in the LCC Box folder.

## Code availability

All other resources and codes used, including information about all the publications related to Figure S2, in this study can be found at 10.5281/zenodo.17102285.
