# Supplementary material for: Soluble immune factor profiles in blood and CSF associated with LRRK2 mutations and Parkinson’s disease
Source: NPJ Parkinsons Dis. 2025 Nov 27;11:365. doi: 10.1038/s41531-025-01215-5 (PMC12749785; doi:10.1038/s41531-025-01215-5)
Supplement: Supplementary file 1 — Supplementary Information [file 41531_2025_1215_MOESM1_ESM.pdf]

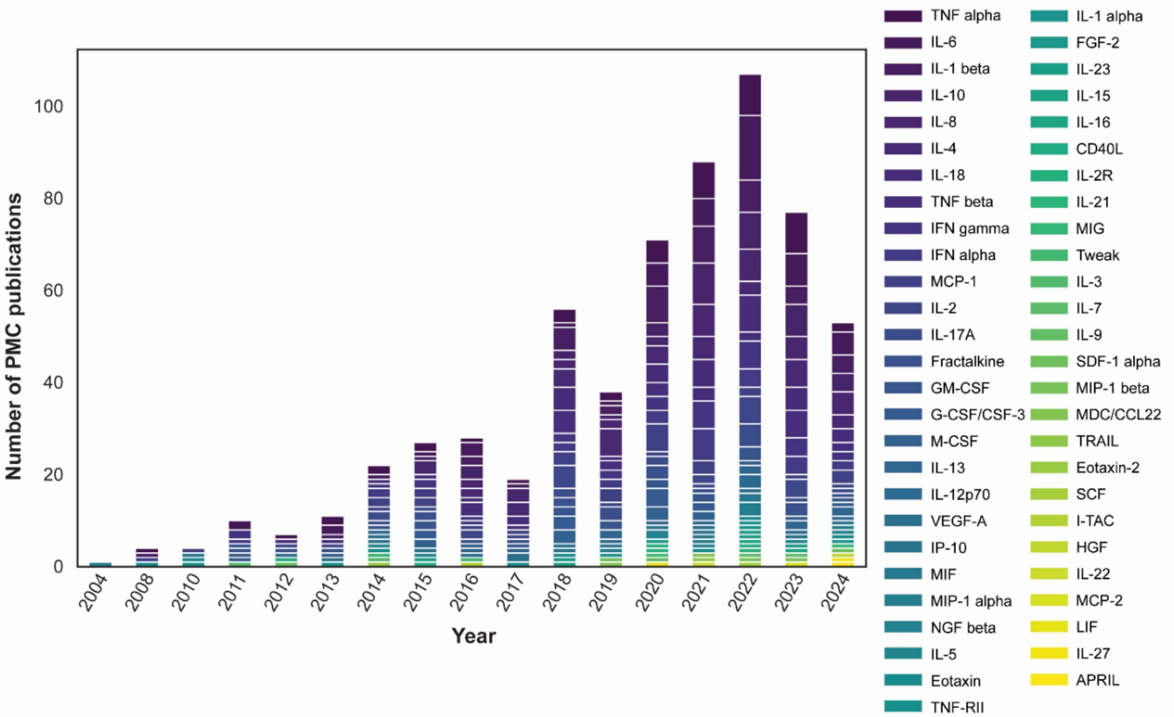

Supplementary Fig 1. The number of PubMed Central (PMC) publications discussing cytokines, *LRRK2*, and Parkinson's disease by publication year. A total of 401 unique papers were retrieved from PubMed and analyzed for 53 cytokines based on keyword search.

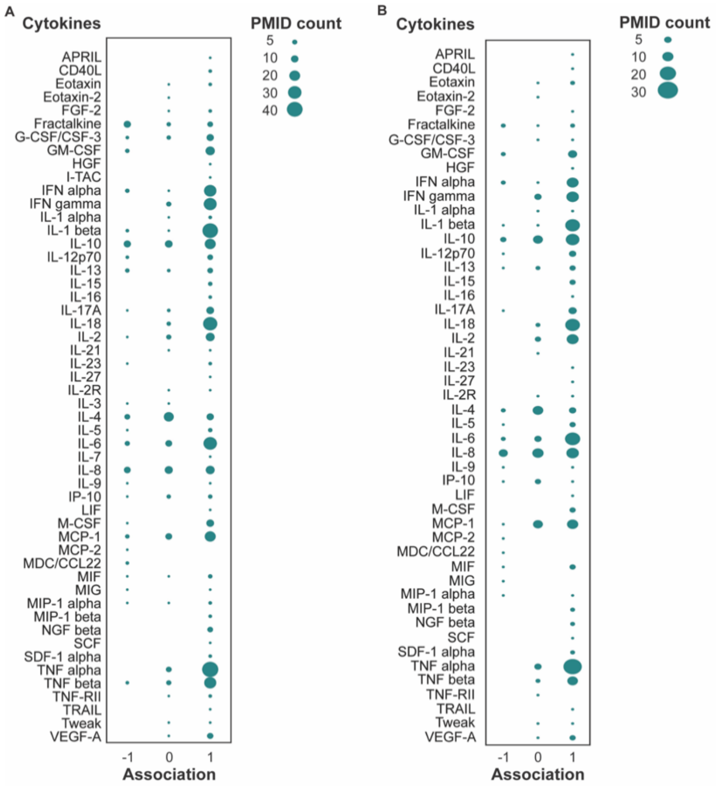

Supplementary Fig 2. GPT-extracted literature overview of cytokines and PD. Scores of 1 were given to positive associations between higher cytokine concentrations and PD, -1 to negative associations between lower cytokine concentrations and PD, and 0 to no associations. Bubble size represents the number of papers supporting the association scores. A: Studies across various hosts, such as humans, animals, and cell lines B: Human studies only. A total of 1060 papers were extracted and filtered for associations with PD or LRRK2, and then a total of 401 papers were used for analysis.

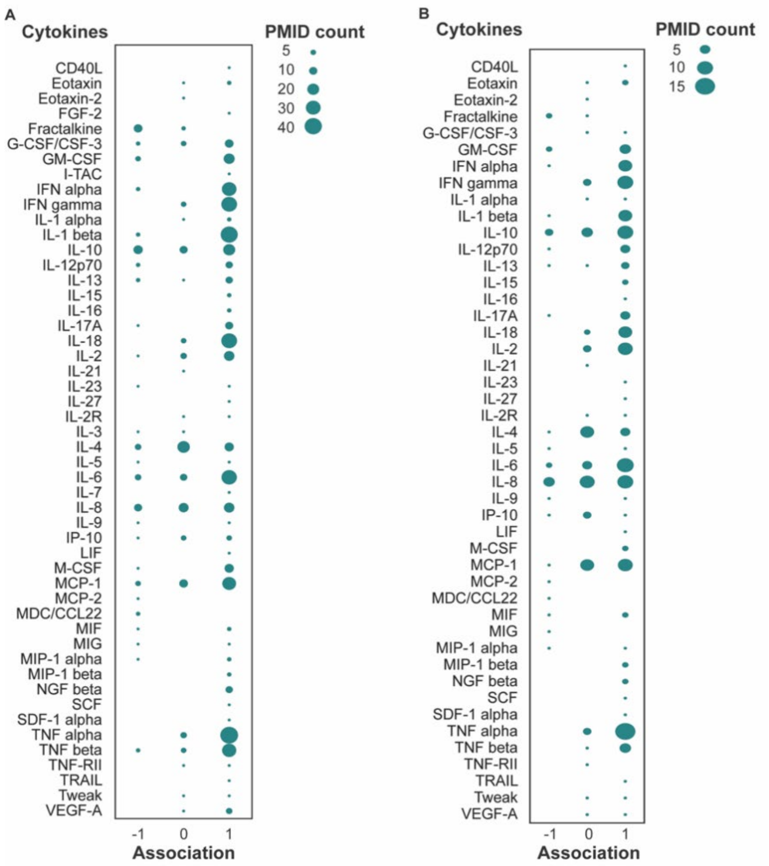

Supplementary Fig 3. GPT-extracted primary literature overview of cytokines and PD. Scores of 1 were given to positive associations between higher cytokine concentrations and PD, -1 to negative associations between lower cytokine concentrations and PD, and 0 to no associations. Bubble size represents the number of papers supporting the association scores. A: Studies across various hosts, such as humans, animals, and cell lines. B: Human studies only. Reviews, editorials, letters, and preprints were excluded, totaling 323 papers (out of 401) that were defined as primary literature used in this figure.

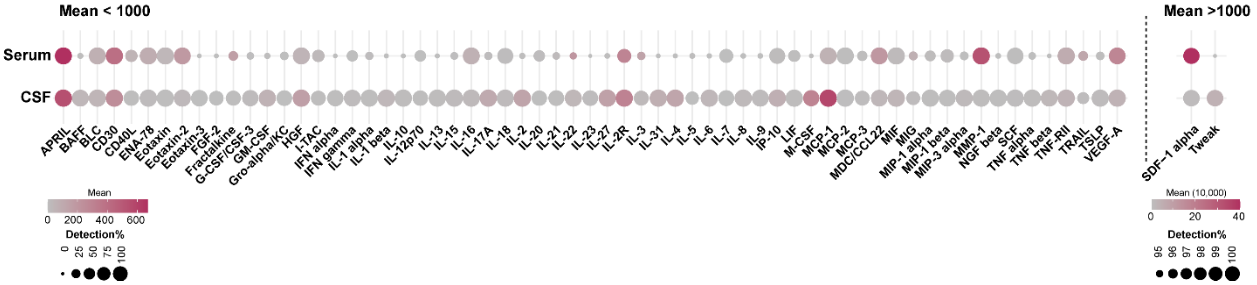

Supplementary Fig 4. Immune factor profiles in serum and CSF. Means of $\log_2$ raw concentration (pg/mL) of soluble immune factors and their detection percentage in serum and CSF samples of the LCC.
